# NHERF1 Enhances Cisplatin Sensitivity in Human Cervical Cancer Cells

**DOI:** 10.3390/ijms18010005

**Published:** 2017-01-12

**Authors:** Tao Tao, Xiaomei Yang, Qiong Qin, Wen Shi, Qiqi Wang, Ying Yang, Junqi He

**Affiliations:** 1 Department of Biochemistry and Molecular Biology, Capital Medical University, Beijing 100069, China; taotaotxwd@ccmu.edu.cn (T.T.); xmyang126@126.com (X.Y.); qqin777@163.com (Q.Q.); shiwen137523@163.com (W.S.); wangqiqi3127@126.com (Q.W.); 2 Beijing International Cooperation Base for Science and Technology on China–UK Cancer Research, Beijing 100069, China; 3 Experiment Center, Beijing Friendship Hospital, Capital Medical University, Beijing 100050, China; 4 Core Facilities Center, Capital Medical University, Beijing 100069, China

**Keywords:** cervical cancer, NHERF1, cisplatin resistance

## Abstract

Cervical cancer is one of the most common female malignancies, and cisplatin-based chemotherapy is routinely utilized in locally advanced cervical cancer patients. However, resistance has been the major limitation. In this study, we found that Na^+^/H^+^ Exchanger Regulatory Factor 1 (NHERF1) was downregulated in cisplatin-resistant cells. Analysis based on a cervical cancer dataset from The Cancer Genome Atlas (TCGA) showed association of NHERF1 expression with disease-free survival of patients received cisplatin treatment. NHERF1 overexpression inhibited proliferation and enhanced apoptosis in cisplatin-resistant HeLa cells, whereas NHERF1 knockdown had inverse effects. While parental HeLa cells were more resistant to cisplatin after NHERF1 knockdown, NHERF1 overexpression in CaSki cells promoted cisplatin sensitivity. Overexpression and knockdown studies also showed that NHERF1 significantly inhibited AKT and extracellular signal–regulated kinase (ERK) signaling pathways in cisplatin-resistant cells. Taken together, our results provide the first evidence that NHERF1 can sensitize cisplatin-refractory cervical cancer cells. This study may help to increase understanding of the molecular mechanisms underlying cisplatin resistance in tumors.

## 1. Introduction

Cervical cancer is the second most common malignancy in women, accounting for an estimated 12,990 new cases and 4120 deaths in the USA for 2016. In the past decade, about one third of patients with invasive cervical cancer died of metastasis [1]. Cisplatin remains the cornerstone in the systemic anti-neoplastic management of locally advanced cervical cancer (LACC). Although the agent has shown efficacy in the treatment of this disease, resistance is a major limitation. The median duration of measurable response is only several months in chemotherapy-naïve patients [2]. Patients with recurrent disease have a poor prognosis with a one-year survival rate between 15% and 20% [3]. Therefore, defining the regulatory mechanisms underlying cisplatin resistance has been an emerging line of research.

Na^+^/H^+^ Exchanger Regulatory Factor 1 (NHERF1) was first identified as an essential cofactor for cyclic adenosine monophosphate (AMP) inhibition of Na^+^/H^+^ exchange in the rabbit renal brush border membrane [4]. It is known to be a scaffold protein highly expressed on the apical aspect of polarized epithelia in many human tissues including kidney, breast, and cervix. NHERF1 contains two N-terminal tandem PSD95/Dlg/ZO-1 (PDZ) domains followed by an ezrin–radixin–moesin (ERM)-binding region [5]. Through these domains, NHERF1 binds to ion transporters, G protein-couple receptors (GPCR), and cytoskeleton-associated proteins, thereby controlling internalization and trafficking of several receptors, ion channels, and transporters [6]. Moreover, NHERF1 has also been shown to interact with cancer-related proteins such as phosphatase and tensin homolog deleted on chromosome ten (PTEN), spleen tyrosine kinase (SYK), platelet-derived growth factor receptor (PDGFR), and epidermal growth factor receptor (EGFR), and modulate their downstream signaling [7,8,9,10]. It has been reported that NHERF1 overexpression reduced activation of AKT and ERK signaling as well as proliferative, migratory, and invasive abilities of both breast cancer cells and lung cancer cells [11,12,13,14]. More importantly, NHERF1 also elicited pharmacologic function by enhancing the response of breast cancer cells to anti-cancer drugs such as geftinib and imatinib [15,16]. These findings suggest a potential role of NHERF1 in chemotherapy.

In the present study, we found that NHERF1 was downregulated in cisplatin-resistant HeLa cells by analysis of a cervical cancer dataset from Gene Expression Omnibus (GEO). We further characterized the regulatory effect of NHERF1 on response of cervical cancer cells to cisplatin and provided a novel clue for the mechanisms underlying cisplatin resistance.

## 2. Results

### 2.1. NHERF1 Expression Is Downregulated in Cisplatin-Resistant HeLa Cells

**Figure 1:**
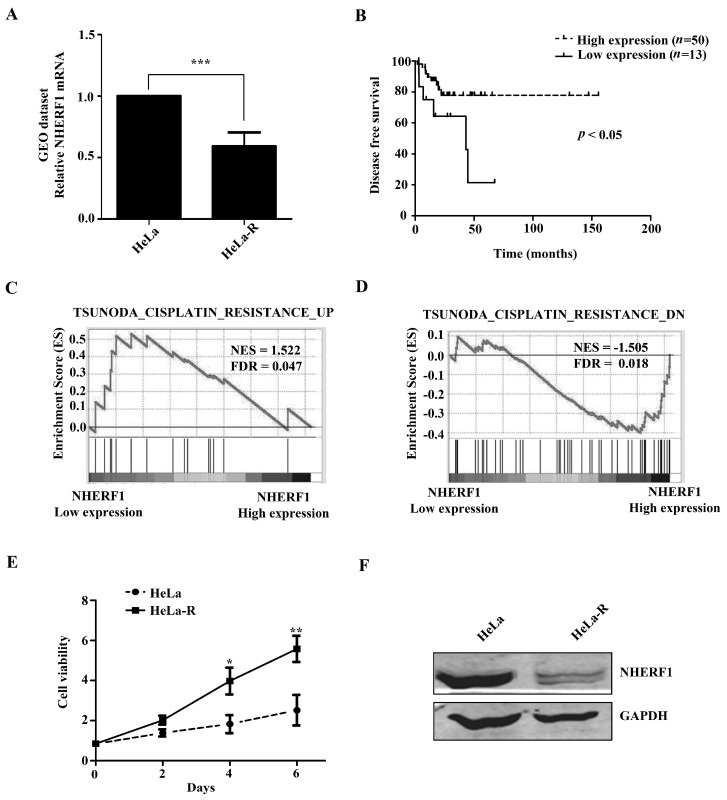
Na^+^/H^+^ Exchanger Regulatory Factor 1 (NHERF1) is downregulated in cisplatin-resistant cervical cancer HeLa cells. (**A**) *NHERF1* gene expression in parental and cisplatin-resistant cervical cancer cells was analyzed based on dataset GSE15120. Data are expressed as fold change compared to parental cells. Results represent the mean ± SD of six samples. *** *p* < 0.001 with respect to parental cells; (**B**) Kaplan-Meier survival analysis of 63 patients with cisplatin treatment in a cervical cancer dataset from TCGA. These patients were divided into high (*n* = 50) and low (*n* = 13) NHERF1 expression groups; (**C**,**D**) GSEA analysis of cervical cancer TCGA dataset based on signatures of cisplatin resistance up-regulated genes (**C**) and cisplatin resistance down-regulated genes (**D**) according to NHERF1 mRNA expression levels; (**E**) Establishment of cisplatin-resistant HeLa cells. Parental and cisplatin-resistant HeLa cells were cultured in the presence of 1.0 μg/mL cisplatin in 96-well plates and stained with cell counting kit-8 (CCK8) at indicated time. Values are presented relative to cell viability on day 0. * *p* < 0.05, ** *p* < 0.01 compared with the parental cells; (**F**) Western blotting analysis of NHERF1 expression in parental and cisplatin-resistant HeLa cells. In (**A**), (**E**), and (**F**), HeLa-R, cisplatin-resistant HeLa cells.

Since cisplatin resistance was generally associated with increased cisplatin export [17], we first analyzed the expression profile of genes annotated to regulation of cell excretion in a dataset GSE15120 from the GEO. The dataset comprises six pairs of parental and cisplatin-resistant HeLa cells. Seven genes were downregulated in all six cisplatin-resistant cells compared with parental cells. Among them, NHERF1 was the most downregulated gene with 40% reduction of mRNA expression level in cisplatin-resistant cells compared with parental cells (Figure 1A and Table 1). To address the role of NHERF1 expression in the prognosis of cervical cancer patients who had been with or without cisplatin treatment, analysis was performed on The Cancer Genome Atlas (TCGA) cervical cancer dataset including 63 patients following cisplatin treatment and 197 patients without cisplatin-treatment history. Tumor characteristics are shown in Table 2. The two cohorts were respectively divided into high NHERF1 expression and low NHERF1 expression groups. Kaplan-Meier survival analysis demonstrated that the high NHERF1 expression group had better outcome than the low NHERF1 expression group in cisplatin-treated patients (median survival 25.3 vs. 15.6 months, *p* < 0.05; Figure 1B). However, no association between disease-free survival and NHERF1 expression level was observed in patients without cisplatin-treatment history (*p* = 0.49; Figure S1). Gene Set Enrichment Analysis (GSEA) on the TCGA cervical cancer dataset showed that the genes related to cisplatin resistance were correlated with NHERF1 expression (Figure 1C,D). These data suggest that NHERF1 expression level may be a cisplatin sensitivity predictor in cervical cancer. To investigate the role of NHERF1 expression in cisplatin resistance, we established cisplatin-resistant HeLa cells, which grew more rapid in the presence of cisplatin (1.0 μg/mL) compared with parental cells (Figure 1E). Western blotting assay confirmed that NHERF1 expression was significantly declined in cisplatin-resistant cells (Figure 1F).

### 2.2. NHERF1 Regulates Cisplatin Sensitivity in Cervical Cancer Cells

**Figure 2:**
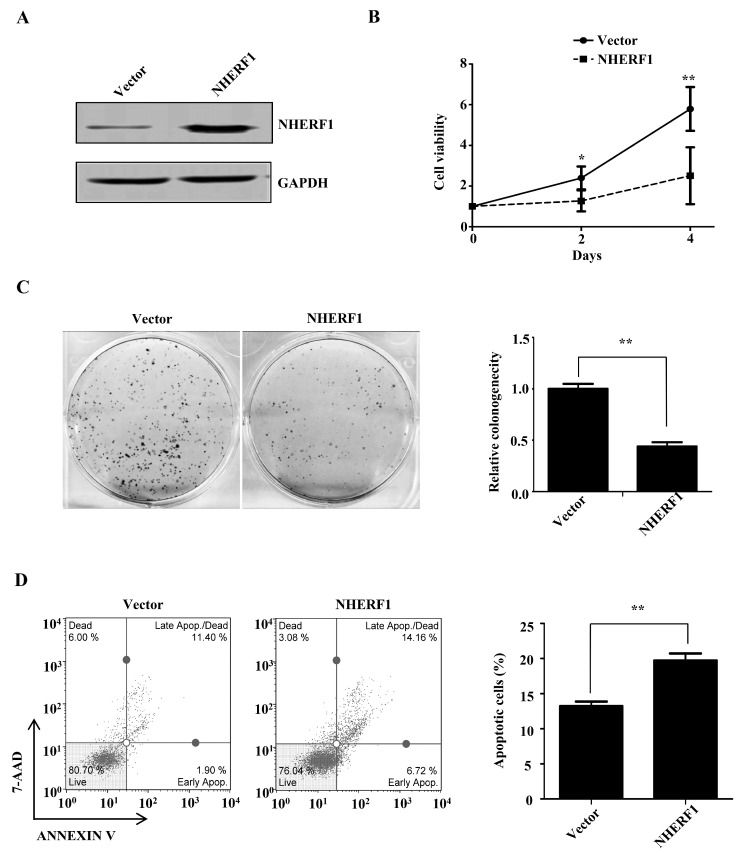
NHERF1 overexpression suppresses cell growth and promotes apoptosis in cisplatin-resistant HeLa cells. (**A**) Western blotting analysis of NHERF1 expression in cisplatin-resistant cells transfected with NHERF1; (**B**–**D**) Effect of NHERF1 overexpression on cell proliferation (**B**), colony formation (**C**), and cell apoptosis (**D**). (**B**) Control vector or NHERF1-transfected cisplatin-resistant HeLa cells were respectively cultured in 96-well plates and stained with CCK8 at 0, 2, 4 days. Growth of control and NHERF1-transfected cells was assessed by measuring absorbance at 450 nm. Values are presented relative to cell viability on day 0. Data represent the mean ± SD of three individual experiments; (**C**) Cells were plated at a concentration of 400 per well. Colonies were photographed (**left panel**) and counted (**right panel**) at 14 days. Values are expressed relative to the colony number formed by cells transfected with control vector. Data represent the mean ± SD of duplicate; (**D**) HeLa cells transfected with either control or NHERF1 were stained with 7-AAD and Annexin V. The percentage of cells in apoptotic phase was quantified with Muse™ Cell Analyser (**left panel**). Data represent the mean ± SD of three individual experiments (**right panel**). * *p* < 0.05, ** *p* < 0.01 compared with vector-transfected cells.

**Figure 3:**
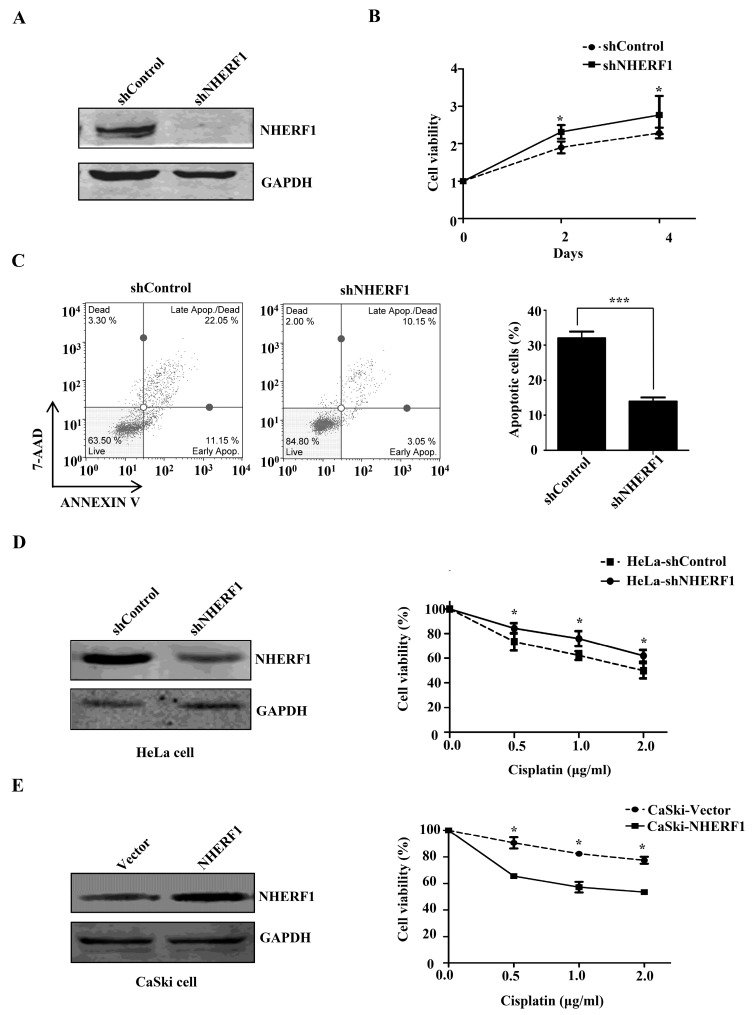
NHERF1 regulates cisplatin sensitivity in cervical cancer cells. (**A**–**C**) Effect of NHERF1 knockdown on cellular function in cisplatin-resistant HeLa cells. (**A**) Western blotting analysis of NHERF1 expression in cisplatin-resistant cells transfected with either control or NHERF1 shRNA; (**B**) Control or NHERF1 shRNA-transfected cisplatin-resistant HeLa cells were respectively cultured in 96-well plates and stained with CCK8 at 0, 2, 4 days. Growth of control and NHERF1 shRNA-transfected cells was assessed by measuring absorbance at 450 nm. Values are presented relative to cell viability on day 0. Data represent the mean ± SD of three individual experiments; (**C**) Cisplatin-resistant HeLa cells transfected with either control or NHERF1 shRNA were stained with 7-AAD and Annexin V. The percentage of cells in apoptotic phase was quantified with Muse™ Cell Analyser (**left panel**). Data represent the mean ± SD of three individual experiments (**right panel**); (**D**) Effect of NHERF1 knockdown on cisplatin sensitivity in parental HeLa cells. shRNA-transfected HeLa cells were incubated for 48 h upon cisplatin treatment; (**E**) Effect of NHERF1 overexpression on cisplatin sensitivity in CaSki cells. Control vector or NHERF1-transfected cells were incubated for 48 h in the presence of cisplatin. In (**D**) and (**E**), left panel: Western blotting analysis of NHERF1 expression. Right panel: Cell viability analyzed by measuring absorbance at 450 nm. Values are presented relative to cell viability in the absence of cisplatin. Data represent the mean ± SD of three individual experiments. * *p* < 0.05, *** *p* < 0.001 compared with control cells.

We next tested whether NHERF1 expression could affect cisplatin sensitivity in cervical cancer cells. Cisplatin-resistant HeLa cells were transfected with NHERF1 and overexpression of NHERF1 protein was confirmed by Western blotting (Figure 2A). Effect of NHERF1 on cell growth was examined by CCK8 colorimetric assay and colony-formation assay. Cell proliferation was significantly suppressed by NHERF1 overexpression in cisplatin-resistant HeLa cells (Figure 2B). Accordingly, NHERF1 overexpression also reduced the ability of colony formation to 50% in cisplatin-resistant HeLa cells as compared with controls (Figure 2C). The effect of NHERF1 on cell apoptosis was assessed by flow cytometry. NHERF1 overexpression led to a significant increase in the proportion of apoptotic cells in cisplatin-resistant HeLa cells (Figure 2D). To verify these results, endogenous NHERF1 expression was abolished by shRNA in cisplatin-resistant cells (Figure 3A), and cell proliferation and apoptosis were evaluated. NHERF1 knockdown significantly increased cell proliferation and decreased apoptotic cells (Figure 3B,C), which is in agreement with the data from the overexpression experiment. To further confirm these findings in cervical cancer cells, NHERF1 was knocked down in HeLa cells and overexpressed in CaSki cells. Cell viability was assessed in the presence of increasing concentrations of cisplatin. While NHERF1-depleted HeLa cells were more resistant to cisplatin than control shRNA-treated population (Figure 3D), NHERF1 overexpression enhanced cisplatin sensitivity of CaSki cells (Figure 3E). Taken together, these data suggest that NHERF1 is involved in the regulation of cisplatin resistance in cervical cancer cells.

### 2.3. NHERF1 Regulates the ERK1/2 and AKT Signaling Pathways in Cisplatin-Resistant Cells

**Figure 4:**
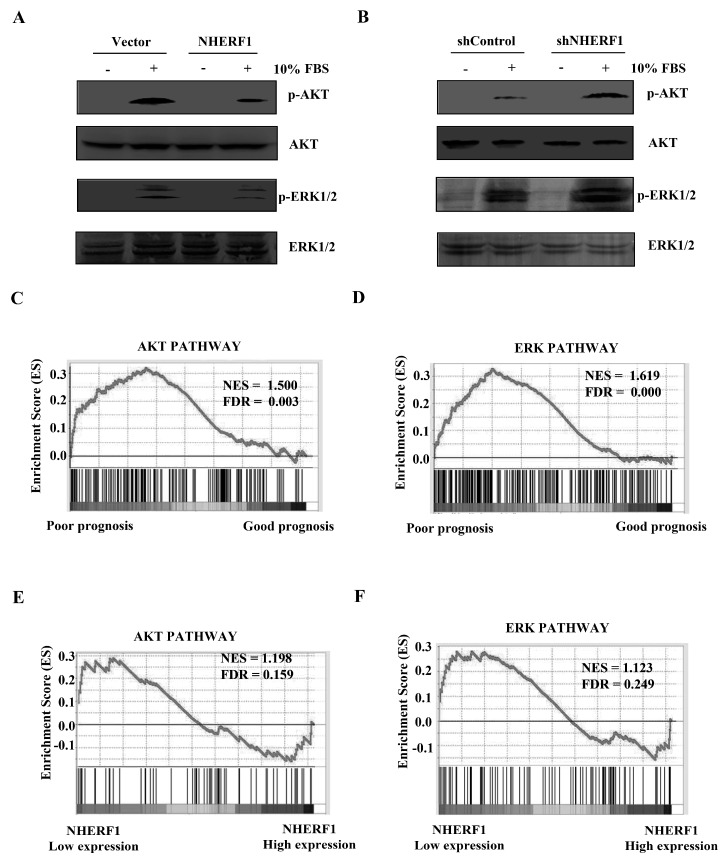
NHERF1 regulates AKT and ERK signaling pathways in cisplatin-resistant cervical cancer cells. (**A**,**B**) Effect of NHERF1 overexpression (**A**) or silencing (**B**) on AKT and ERK signaling. Transfected cells were serum starved for 16 h before stimulation with 10% FBS for 15 min. Phosphorylation of AKT or ERK was analyzed by Western blotting with anti-*p*-AKT or anti-*p*-ERK antibodies, respectively. The blot was reprobed with anti-AKT or anti-ERK antibodies as loading control. The results are representative of three independent experiments; (**C**,**D**) Enrichment plots of gene expression signatures for the AKT (**C**) or ERK (**D**) pathway according to clinical outcome by GSEA of TCGA cervical cancer database; (**E**,**F**) Enrichment plots of gene expression signatures for AKT (**E**) or ERK (**F**) pathway according to NHERF1 mRNA expression levels by GSEA of cervical cancer databases GSE15120. Data from cisplatin-resistant cells were divided into high and low NHERF1 expression groups.

The activation of ERK1/2 and AKT signaling has been implicated in cisplatin resistance [17]. In order to determine whether NHERF1 could affect these signaling pathways, NHERF1 was overexpressed or knocked down in cisplatin-resistant HeLa cells. After stimulation with 10% fetal bovine serum (FBS) for 15 min, the levels of phosphorylated ERK1/2 and AKT were examined. NHERF1 overexpression significantly inhibited phosphorylation of both ERK1/2 and AKT (Figure 4A), whereas the activity of both signaling pathways was increased upon NHERF1 knockdown (Figure 4B). To delineate the relevance of AKT and ERK1/2 activation with outcome in cisplatin-treated cervical cancer patients, the patients in TCGA dataset were divided into two groups according to disease-free survival (DFS) time: good prognosis (DFS time > 3 years) and poor prognosis groups (DFS time < 3 years). GSEA data showed that gene signatures in AKT or ERK1/2 signaling pathways were enriched in the poor prognosis group (Figure 4C,D). To further investigate the correlation of NHERF1 expression with AKT or ERK1/2 signaling in cisplatin-resistant cells, the data in the dataset GSE15120 were divided into high NHERF1 expression and low NHERF1 expression groups. GSEA results showed that the gene signatures of AKT and ERK target genes were highly enriched in the low NHERF1 expression group (Figure 4E,F).

## 3. Discussion

In the present study, we first found that both mRNA and protein expression levels of NHERF1 are downregulated in cisplatin-resistant HeLa cells as compared with parental cells. Modulation of NHERF1 expression in cisplatin-resistant HeLa cells by overexpression or shRNA could alter cell proliferation, ability to colony formation, and cell apoptosis. Downregulation of NHERF1 expression in HeLa cells significantly increased cisplatin resistance. We also showed that NHERF1 regulates the activation of AKT and ERK signaling in cisplatin-resistant cells. Our data suggest that NHERF1 may enhance cisplatin chemosensitivity in cervical cancer.

Mounting evidence has demonstrated that cisplatin resistance occurs mainly by defect in apoptotic signaling and change in cellular accumulation of cisplatin [17]. It has been shown that cisplatin-induced DNA damage triggered phosphorylation of Bcl-2-associated agonist of cell death (BAD) through AKT and ERK signaling cascades. BAD phosphorylation is required for cell viability in cisplatin-resistant cells [18]. The inhibition of either of the two signaling pathways could sensitize many cancer cells to cisplatin [19,20,21]. We and other groups reported that NHERF1 could form signaling complexes with PTEN or tyrosine kinase receptors including PDGFR and EGFR to counterbalance AKT and ERK signaling in breast cancer cells [7,11]. Similar results were also observed in cisplatin-resistant HeLa cells (Figure 4). Hence, it is possible that NHERF1 might regulate cisplatin resistance through the inhibition of anti-apoptotic signaling.

On the other hand, NHERF1 belongs to a superfamily of PDZ proteins which have been implicated in regulating the membrane abundance, localization, and function of a number of drug transporters [6]. NHERF1 was annotated to regulation of cell excretion in Gene Ontology (Table 1). Indeed, previous studies indicated the functional relevance of NHERF1 with multidrug resistance protein 4 (MRP4), a member of the ATP-binding cassette transporter superfamily. Increased expression of MRP4 in ovarian cancer cells led to resistance to cisplatin with significantly reduced accumulation of cisplatin [22]. Consistently, Zhang et al. reported that MRP4 knockdown reversed cisplatin resistant phenotype of gastric cancer cells [23]. NHERF1 can interact directly with MRP4. Downregulation of NHERF1 by siRNA in HeLa cells markedly increased MRP4 level at the plasma membrane, thereby enhancing efflux function [24]. Meanwhile, we found an inverse correlation between expression levels of NHERF1 and MRP4 through analysis of TCGA cervical cancer dataset (Figure S2), suggesting an inhibitory effect of NHERF1 on MRP4 expression. Given these findings, it is tempting to speculate that NHERF1 expression might increase cisplatin accumulation by regulating expression and internalization of MRP4. Further studies are required to confirm this hypothesis.

Downregulation of NHERF1 was found in cisplatin-resistant cells (Figure 1). By virtue of the fact that NHERF1 could enhance the cisplatin sensitivity, changed NHERF1 expression might be due to colony selection in which cells with low NHERF1 expression are more resistant to cisplatin than those with high NHERF1 expression. In conclusion, our study provides the first evidence that NHERF1 can regulate sensitivity of cervical cancer cells to cisplatin, which offers more insights into the molecular mechanism underlying cisplatin resistance.

## 4. Materials and Methods

### 4.1. Datasets Collection

The microarray data for parental and cisplatin-resistant cells were downloaded from the Gene Expression Ominibus (GEO, available online: http://www.ncbi.nlm.nih.gov/geo/) public database under accession number GSE15120. The dataset GSE15120 consists of six pairs of parental and cisplatin-resistant HeLa cell lines. The TCGA Cervical Carcinoma mRNA expression (RNA seq V2) data were downloaded from Snapage Dataset (available online: http://www.snapage.org; syn1461153) and corresponding clinical data from the cBioPortal for Cancer Genomics (available online: http://cbioportal.org). All the data were processed using standard methods. The genes annotated to regulate excretion were analyzed by Gene Ontology (available online: http://geneontology.org/).

### 4.2. Gene Set Enrichment Analysis

The association between expression of NHERF1 and biological processes was analyzed using Gene Set Enrichment Analysis (GSEA v2.0, available online: http://www.broad.mit.edu/gsea/). GSEA calculates a pathway Enrichment Score (ES) that estimates whether genes from a pre-defined gene set of target genes related to cisplatin resistance (TSUNODA_CISPLATIN_RESISTANCE_DN and TSUNODA_CISPLATIN_RESISTANCE_UP) and AKT/ERK pathway (AKT_UP.V1_UP and GO_REGULATION_OF_ERK1_AND_ERK2_CASCADE) (available online: http://software.broadinstitute.org/gsea/msigdb/index.jsp) are enriched among the highest- (or lowest-) ranked genes or distributed randomly. Default settings were used. Thresholds for significance were determined by permutation analysis (1000 permutations). False Discovery Rate (FDR) was calculated. A gene set is considered significantly enriched when the FDR score is <0.25.

### 4.3. Cell Culture and Establishment of Cisplatin-Resistant HeLa Cells

The human cervix carcinoma HeLa and CaSki cell lines were obtained from the American Type Culture Collection (Manassas, VA, USA). Cells were maintained in Dulbecco’s modified Eagle’s medium (DMEM, Gibco, Gaithersburg, MD, USA) containing 10% FBS (Gibco), 100 μg/mL streptomycin, and 100 units/mL penicillin and grown at 37 °C in a humidified atmosphere of 5% CO_2_. For establishment of cisplatin-resistant cell line, HeLa cells were continuously cultured in the presence of increasing concentrations (0.1–1.0 μg/mL) of cisplatin (QILU Pharmaceutical, Jinan, China) for 3 months. Cisplatin-resistant HeLa cells were cultured in the presence of 1.0 μg/mL cisplatin. Cisplatin was prepared as stock solution of 1 mg/mL in 0.9% NaCl solution and stored at −20 °C.

### 4.4. Antibodies

Anti-NHERF1 monoclonal antibody (1:1000 working dilution) from BD Biosciences (San Jose, CA, USA). Anti-NHERF1 polyclonal antibody, anti-AKT polyclonal antibody, anti-pS473AKT monoclonal antibody, and anti-pERK monoclonal antibody (1:1000 working dilution) were purchased from Cell Signaling Technology (Danvers, MA, USA). Anti-ERK antibody polyclonal antibody (1:1000 working dilution) from Upstate Biotechnology (Lake Placid, NY, USA). Anti-GAPDH (glyceraldehyde-3-phosphate dehydrogenase) monoclonal antibody (1:2000 working dilution) and horseradish peroxidase (HRP)-conjugated secondary antibody were obtained from ZSGB-BIO (Beijing, China).

### 4.5. Plasmids and Transfection

Hemagglutinin (HA)-tagged full-length NHERF1 were kindly provided by Randy Hall (Emory University, Atlanta, GA, USA). pSuper.puro luciferase control (shNC) plasmid and pSuper.puro shNHERF constructs were kind gifts from Margaret J. Wheelock (University of Nebraska Medical Center, Omaha, NE, USA). All of the DNA constructs were individually verified by DNA sequencing.

Next, 3 × 10^5^ cells were seeded into a six-well plate and then transfected with 1 μg plasmids using Lipofectamine 2000 (Invitrogen, Carlsbad, CA, USA) following the manufacturer’s instructions.

### 4.6. Western Blotting

Cells were washed with ice-cold phosphate-buffered saline (PBS, pH 7.4) and lysed in ice-cold HNTG buffer (50 mmol/L HEPES (pH = 7), 50 mmol/L NaF, 1 mmol/L EGTA, 150 mmol/L NaCl, 1% Triton X-100, 10% glycerol, 1.5 mmol/L MgCl_2_) containing a mixture of protease inhibitors (Roche Applied Science, Indianapolis, IN, USA). Proteins were separated by SDS-Polyacrylamide Gel Electrophoresis (SDS-PAGE) and then transferred to a nitrocellulose membrane. The membrane was blocked in TBST (20 mM Tris/HCl, pH 7.5, 500 mM NaCl and 0.1% Tween 20) containing 5% (*w*/*v*) non-fat dried milk for 1 h at room temperature. Proteins of interest were probed with corresponding primary antibodies followed by HRP (horseradish peroxidase)-conjugated anti-rabbit or anti-mouse secondary antibodies. Immunoreactive bands were visualized by enhanced chemiluminescence (ECL) detection reagents (Applygen Technologies, Beijing, China) and Odyssey infrared imaging system (LI-COR Biosciences, Lincoln, NE, USA), respectively. For cell signaling study, cells were transfected with indicated plasmids and cultured for 36 h. Then the cells were starved in serum-free medium for 16 h before stimulation for 15 min with 10% FBS.

### 4.7. Cell Viability Assay

Cells were cultured in 96-well microplates at a density of 3000 cells/well. NHERF1 overexpression or shRNA-transfected cisplatin-resistant cells were seeded in DMEM containing 10% FBS 24 h post-transfection. At indicated time after plating, CCK-8 (Dojindo, Kumamoto, Japan) was added to each well and the cells were cultured for another 1 h. Viable cells were quantified by measuring absorbance at 450 nm with an EnVision multilabel reader (PerkinElmer, Waltham, MA, USA).

### 4.8. Cell Apoptosis Assay

For the cell apoptosis assay, 1 × 10^6^ cells were harvested 48 h post-transfection, and then labeled for 20 min at 37 °C in the dark with the Muse™ Annexin-V & Dead Cell reagent (Merck-Millipore, Darmstadt, Germany) according to manufacturer’s instructions. A total of 4000 events were collected and analyzed using the Muse™ Cell Analyser (Merck-Millipore).

### 4.9. Colony Formation Assay

Cisplatin-resistant cells were transfected with control vector or NHERF1 and cultured for 24 h. Then, the cells were seeded in a six-well plate at a density of 400 cells/well and cultured with 1.0 μg/mL cisplatin for two weeks. After three washes with PBS, cells were fixed for 15 min in 100% methanol, and then stained for 25 min with 0.5% crystal violet (Sigma-Aldrich, St. Louis, MO, USA). Colonies of more than 50 cells were counted using an inverted microscope (Olympus CKX41; Olympus Corp., Tokyo, Japan). Colony efficiency (CE) and the rate of colony inhibition (CI) were calculated as follows: CE = (colonies formed/cells seeded) × 100% and CI = (CE in experimental group/CE in control group) × 100%, respectively.

### 4.10. Statistical Analysis

Statistical analyses were performed using the GraphPad Prism 5 software (La Jolla, CA, USA) and SPSS 18.0 (SPSS Inc, Chicago, IL, USA). All data were presented as means ± SD and statistical significance was analyzed by independent sample *t*-test. Differences were considered significant when *p* < 0.05. Each assay was repeated at least three times.

## Figures and Tables

**Table 1:** Expression profile of genes annotated to regulation of excretion in cisplatin-resistant cells.

Gene	Log (FC)
*NHERF1*	−0.78205
*TAC1*	−0.72346
*AGTR1*	−0.53629
*AVPR1A*	−0.45374
*COMT*	−0.44788
*EDN1*	−0.35517
*PTGER3*	−0.23476

**Table 2:** Clinical characteristics of cervical cancer patients in TCGA dataset.

Patient and Tumor Characteristics	*N* (%)
Age (years)	
Mean	47
Range	20–88
Histological type	
Squamous carcinoma	231 (89)
Adenocarcinoma	29 (11)
Chemotherapy	
Cisplatin	63 (24)
None	197 (76)
Disease-free survival (DFS) status	
Disease Free	213 (82)
Recurred/Progressed	47 (18)

## Supplementary Materials

Supplementary materials can be found at www.mdpi.com/1422-0067/18/1/5/s1.

## Abbreviations

NHERF1	Na^+^/H^+^ Exchanger Regulatory Factor 1
LACC	Locally advanced cervical cancer
ERM	Ezrin–radixin–moesin
GPCR	G protein-couple receptor
GEO	Gene Expression Omnibus
TCGA	The Cancer Genome Atlas
AMP	Adenosine Monophosphate
PDZ	PSD95/Dlg/ZO-1
CCK8	Cell Counting Kit-8
FBS	Fetal bovine serum
BAD	BCL2 Associated Agonist Of Cell Death
FDR	False Discovery Rate
DMEM	Dulbecco’s modified Eagle’s medium
GAPDH	Glyceraldehyde-3-phosphate dehydrogenase
PTEN	Phosphatase and tensin homolog deleted on chromosome ten
SYK	Spleen tyrosine kinase
PDGFR	Platelet-derived growth factor receptor
EGFR	Epidermal growth factor receptor
ERK	Extracellular signal–regulated kinase
SDS-PAGE	SDS-Polyacrylamide Gel Electrophoresis
