# Supplementary material for: NHERF1 Enhances Cisplatin Sensitivity in Human Cervical Cancer Cells
Source: Int J Mol Sci. 2017 Jan 12;18(1):5. doi: 10.3390/ijms18010005 (PMC5297640; doi:10.3390/ijms18010005)
Supplement: Supplementary file 1 [file ijms-18-00005-s001.pdf]

Supplementary Materials: NHERF1 Enhances Cisplatin Sensitivity in Human Cervical Cancer Cells

Tao Tao, Xiaomei Yang, Qiong Qin, Wen Shi, Qiqi Wang, Ying Yang and Junqi He

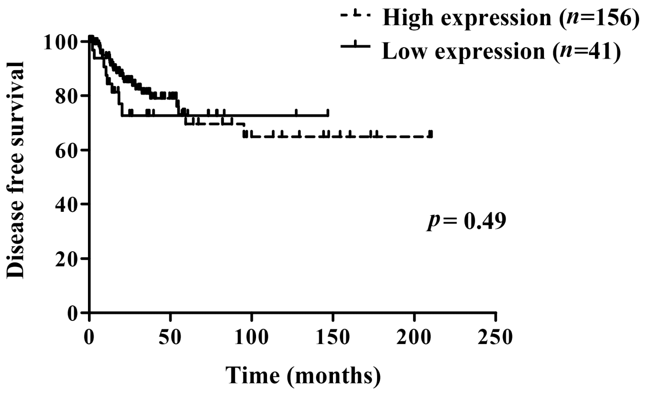

Figure S1. Kaplan-Meier survival analysis of cervical cancer TCGA dataset from 197 patients without cisplatin treatment.

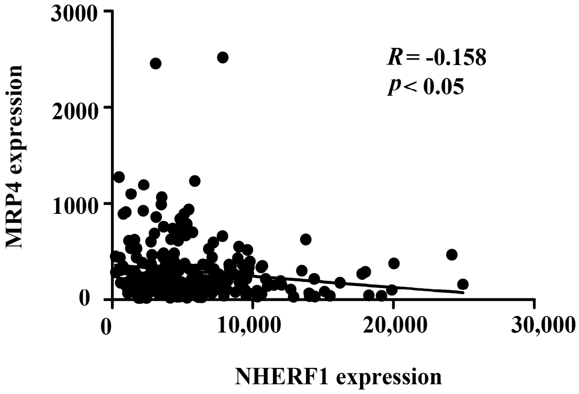

Figure S2. NHERF1 expression is negatively associated with MRP4 expression in the cervical cancer TCGA dataset.
